# Neutrophil activity in sepsis: a systematic review

**DOI:** 10.1590/1414-431X20207851

**Published:** 2020-10-21

**Authors:** C.B. Resende, I. Borges, W.A. Gonçalves, R. Carneiro, B.M. Rezende, V. Pinho, V. Nobre, M.M. Teixeira

**Affiliations:** 1 Hospital das Clínicas, Universidade Federal de Minas Gerais, Belo Horizonte, MG, Brasil; 2 Núcleo Interdisciplinar de Investigação em Medicina Intensiva, Belo Horizonte, MG, Brasil; 3 Departamento de Clínica Médica, Faculdade de Medicina, Universidade Federal de Minas Gerais, Belo Horizonte, Minas Gerais, Brasil; 4 Instituto de Ciências Biológicas, Universidade Federal de Minas Gerais, Belo Horizonte, MG, Brasil; 5 Departamento de Enfermagem Básica, Escola de Enfermagem, Universidade Federal de Minas Gerais, Belo Horizonte, Minas Gerais, Brasil

**Keywords:** Neutrophil, Sepsis, Neutrophil recruitment, Neutrophil activation, Systematic review

## Abstract

The neutrophil is an important cell in host defense against infections, acting as the first line of microorganism control. However, this cell exhibits dysregulated activity in sepsis and may contribute to the pathogenesis of the disease. This systematic review aimed to highlight the major scientific findings regarding neutrophil activity in sepsis reported in clinical and experimental research published in the last 10 years. The search was conducted in the Virtual Health Library of PAHO-WHO (BVS) and PubMed databases, and articles published between January 2007 and May 2017 in Portuguese, English, and Spanish were eligible. Article selection was carried out independently by two reviewers (CB and IB). A total of 233 articles were found, of which 87 were identified on PubMed and 146 on BVS. Eighty-two articles were duplicates. Of the remaining 151 articles, 19 met the inclusion criteria after title, abstract, and full-text analysis. Overall, research in clinical samples and animal models of sepsis showed reduced capacity of neutrophils to migrate and delayed apoptosis, but there was no consensus on the phagocytic activity of neutrophils in sepsis. Molecules, such as pentraxin 3 (PTX3), have been analyzed as potential diagnostic markers in sepsis but the diversity of soluble molecules detected in blood samples of sepsis patients did not enable further understanding of the correlation of these circulating molecules with neutrophil activity during sepsis. Optimal understanding of the function of neutrophils in sepsis remains a challenge that, if overcome, would eventually allow targeted therapeutic interventions in patients affected by this severe syndrome.

## Introduction

Sepsis is defined as a dysregulated host response to an infectious agent, with dysfunction of one or more organ systems. Organic dysfunction in sepsis is revealed by different signs, such as tissue hypoperfusion, lactic acidosis, oliguria, and acute changes in cerebral function, including acute confusion (1,2).

Sepsis is a cascade of events originating from innate and adaptive immune responses; it is characterized by the activation of various cell types and release of both pro-inflammatory and anti-inflammatory molecules. In the initial phase of sepsis, a predominantly hyperinflammatory state caused by cellular interactions with the infectious agent develops, followed by a state of immune hyporesponsivity (2,3).

Neutrophils are the first cells to migrate through the vascular epithelium and reach the site of infection (4). The chemokines CXCL1, CXCL2, leukotriene B4, and interleukin-8 (CXCL8) released during this stage act on leukocyte rolling by inducing stable binding between adhesion molecules and generating a concentration gradient that stimulates the transmigration of neutrophils from the bloodstream to the site of infection (5).

Evidence shows that, in sepsis, the neutrophil response may be inadequate to eliminate the infectious agent (6–10). Bacteria or their by-products induce disseminated activation of toll-like receptors (TLR-2, TLR-4, and TLR-9) and the subsequent exaggerated release of cytokines and chemokines into the bloodstream (7 [Bibr B08] [Bibr B09]). These mechanisms result in hyperinflammation, which alters neutrophil function in sepsis.

Changes in the profile of neutrophil response in sepsis have been the focus of various studies. For example, the identification of alterations in cellular signaling pathways could serve as the basis for targeted drug development aimed to treat this condition. Accordingly, this systematic review aimed to highlight the major scientific findings regarding neutrophil activity in sepsis based on clinical and experimental research published in the last 10 years.

## Material and Methods

A search on the BVS database (Virtual Health Library of PAHO-WHO) was performed using the following descriptors and format: ((“Neutrophil Activation” OR “Activación Neutrófila” OR “Ativação de Neutrófilo” OR “Neutrophil Infiltration” OR “Infiltración Neutrófila” OR “Infiltração de Neutrófilos” OR “Células LE” OR “Leucócitos Polimorfonucleares” OR “LE cells” OR “Polymorphonuclear leukocytes”)) OR (ti: (Neutrophils OR Neutrófilos)) OR (mh: (Neutrophils OR Neutrófilos))) AND ((ti: (Sepsis OR Sepse OR Sepsia OR Sépsis OR Septicemia OR Piemia OR Pioemia )) OR (mh: (Sepsis OR Sepse OR Sepsia OR Sépsis OR Septicemia OR Piemia OR Pioemia )))) AND ((ti: (Biomarkers OR Biomarcadores OR Marcadores OR Experimental OR Bookmark)) OR (tw: (“Clinical Trial” OR “Ensayo Clínico” OR “Ensaio Clínico” OR “Clinical Study” OR “Estudio Clínico” OR “Estudo Clínico” OR “Estudo de Intervenção” OR “Estudos de Intervenção” OR “Estudio de Intervención” OR “Estudios de Intervención” OR “Intervention Study” OR “Intervention Studies”)) OR (mh: (Biomarkers OR Biomarcadores OR Marcadores OR Experimental OR Bookmark)))) AND (la: (“en” OR “es” OR “pt”).

For the PubMed search, the following descriptors were used: (((((((“Neutrophil Activation”[Title/Abstract] OR “Neutrophil Infiltration”[Title/Abstract])) OR “Neutrophils”[Title]) OR (((“Neutrophil Activation”[Mesh: noexp]) OR “Neutrophil Infiltration”[Mesh: noexp]) OR “Neutrophils”[Mesh:noexp]))) AND (((“Sepsis”[Title] OR Septicemia[Title] OR Piemia[Title] OR Pioemia[Title])) OR “Sepsis”[Mesh: noexp]))) AND ((((“Clinical Trial”[Title/Abstract] OR “Clinical Study” [Title/Abstract])) OR “Biomarkers”[Title]) OR (((“Biomarkers”[Mesh: noexp]) OR “Clinical Trial”[Publication Type: noexp]) OR “Clinical Study”[Publication Type: noexp])).

Studies published between January 2007 and May 2017 in Portuguese, English, and Spanish were included. Study selection was carried out by two independent reviewers (CB and IB) after discussion and agreement of the following eligibility criteria: original articles; neutrophil function clearly identified by name, function, or any direct mention regarding the analysis of this cell; a clear mention of direct neutrophil activity in the context of clinical or laboratory sepsis in the abstract; and finally, studies with descriptions of neutrophil activity in clinical or laboratory sepsis. In every stage of analysis, disagreement between reviewers pertaining to the inclusion or exclusion of specific studies was resolved by a third reviewer. Guideline and review studies were excluded.

## Results

### Description and characteristics of included studies

**Figure 1:**
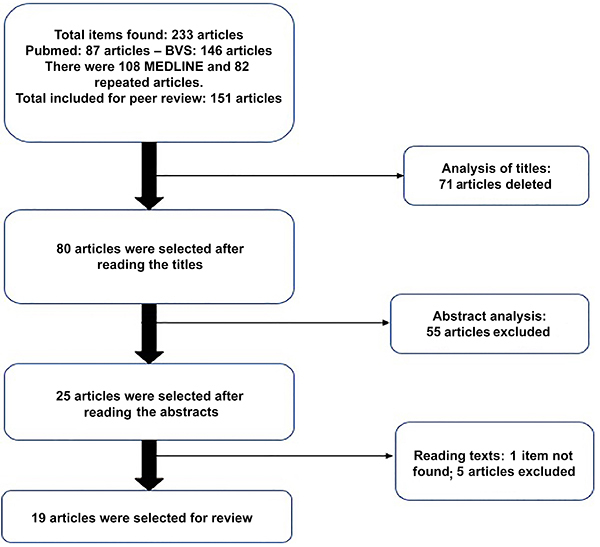
Description of the peer review and selection process of articles.

A total of 233 articles were found, of which 87 were identified on PubMed and 146 on BVS. Eighty-two articles were duplicates. The remaining 151 articles were submitted to title review, during which 71 studies were excluded for not directly describing neutrophils or neutrophil activity. In the abstract review stage, 55 studies we further excluded, and in the full-text review, another five articles were excluded for not fulfilling the criteria of direct analysis of neutrophil activity in sepsis. In addition, one study was not available for full-text review and therefore excluded. A total of 214 studies were excluded in all stages combined. Thus, after independent review by both parties, a total of 19 articles that fulfilled all aforementioned items were selected (Figure 1).

Studies were initially grouped into 2 experimental studies, 3 clinical and experimental studies, 12 clinical studies, and 2 clinical trials. All selected studies were published in English (Supplementary Table S1).

**Figure 2:**
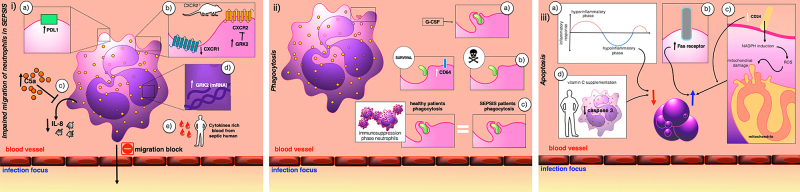
Neutrophil activity in sepsis: chemotaxis, phagocytosis, and apoptosis. Figure created from the results obtained in this review.**Panel i**: Description of the main mechanisms related to neutrophil chemotaxis in sepsis. a) Increased PDL1 was associated with reduced chemotaxis; b) CXCR2 receptor internalization occurs when GRK2 has increased expression resulting in chemotaxis drop. CXCR1 internalization was associated with a drop in neutrophil chemotaxis; c) Increased blood C5a was associated with blockade of chemotactic stimulation exerted by IL-8 on neutrophils; d) Increased GRK2 expression in chemotaxis-depleting neutrophils; e) Septic patients have increased cytokines in their blood (11–20). **Panel ii**: Description of the main mechanisms related to neutrophil phagocytosis in sepsis. a) Growth factor granulocyte stimulating factor (G-CSF) was associated with increased neutrophil phagocytosis; b) Patients who survived the septic event had a higher CD64 expression than patients who died; c) In the immunosuppression phase of sepsis, phagocytosis occurred in the same proportion as healthy volunteers (11,17,24). **Panel iii:** Description of the main mechanisms related to neutrophil apoptosis in sepsis. a) In the hyporesponsiveness phase of sepsis, there is a reduction in neutrophil apoptosis; b) Increased Fas receptor expression was associated with increased neutrophil apoptosis; c) Reduction of CD24 receptor expression was associated with increased neutrophil apoptosis through NADPH induction and consequent mitochondrial damage; d) Patients receiving vitamin C had lower caspase 3 expression (13 [Bibr B14],^17^,^21^–^23^).

Full-text analysis revealed that the studies focused on analyzing migration capacity, phagocytosis, or apoptosis (Figure 2). In both experimental and clinical studies, the authors sought to identify cellular membrane molecules, signaling pathways, or treatments that could alter neutrophil activation pathways.

### Types of tests performed across studies

#### Neutrophilic migration and chemotaxis

Nine articles analyzed neutrophil migration focusing on evaluating the presence or absence of molecules involved in the direct modification of neutrophil migration capacity or treatment and analysis of soluble plasma molecules indirectly related to neutrophil migration. All studies detected a reduction in migratory capacity in cells derived from animal experimental models of sepsis or patients with sepsis (11–15).

In molecular analyses, the role of granulocyte colony-stimulating factor (G-CSF) was highlighted, showing the reduction of neutrophil migration as well as improvement in the immune response of rats subjected to peritonitis and treated with this factor (11). G-CSF was therefore associated with improvement in the immune response of neutrophils in severe infection (11).

Molecules known to participate in the mechanism of neutrophil migration, such as GRK2 and CXCR2, were shown to be increased and decreased, respectively, in neutrophils obtained from experimentally-induced sepsis by cecal ligation and puncture (CLP) (12). Another study that analyzed samples of sepsis patients showed low GRK2 mRNA expression, a finding that was indirectly associated with a reduction in granulocyte migration (19).

Type I and type IV toll-like receptors (TLRs) were shown to induce CCR2 expression in neutrophils from septic humans and animal CLP models, conferring to these cells the ability to migrate in response to CCL2 ligands (10). The same study showed, in neutrophils obtained from CLP animals, that suppression of NFκB was associated with a reduction of neutrophil migration through negative regulation of CCR2 (15).

Additionally, Wang et al. (13) showed that the molecule programmed death ligand (PD-L1) was overexpressed in neutrophils with specific phenotypes (low levels of CD11a, CD62L, and CCR2 and high levels of CD16 and CD64) that showed impaired migration in both CLP animal models and human patients. As such, the authors concluded that sepsis induces the expression of PD-L1, which, in turn, compromises neutrophil chemotaxis.

In a study carried out by Xu et al. (16), a defect in neutrophil migration was identified and associated with the detection of circulating C5a in samples obtained from sepsis patients, raising the possibility that production of C5a induces a reduction of IL-8 secretion by neutrophils, which results in neutrophil dysfunction.

Another line of investigation was the indirect analysis of neutrophil migration capacity by investigating the molecules involved in adhesion and rolling. The study of Blom et al. (18) showed that neutrophils exposed to plasma obtained from patients in the acute phase of sepsis, which is rich in predominantly pro-inflammatory cytokines, had altered mechanisms of adhesion via β_2_-integrins and ICAM-1.

One of the clinical trials included in this review analyzed the effect of lidocaine on cellular recruitment (20). Lidocaine was associated with the control of cellular adhesion induced by chemokines and of neutrophil transmigration (20). Thus, the authors suggested that lidocaine may have a therapeutic effect in hyperinflammatory phases of sepsis by blocking cellular recruitment and adherence.

A study by Demaret et al. (17) was the only one to evaluate neutrophil function in the late phase of sepsis. By comparing cells obtained from sepsis patients and healthy volunteers, the authors identified that in this phase there is also a reduction of neutrophil migration capacity. Despite the increased cell influx in this phase, neutrophils were shown to be immature, with low expression of CXCR1 and CXCR2 (17).

#### Apoptosis

The occurrence of neutrophil apoptosis in sepsis was investigated in five (26%) of the studies included in this review. In all of those studies, the authors observed reduced expression of cellular apoptosis markers in samples from sick humans and experimental models of sepsis (13,17,).

The main focus across studies was identifying molecules or cells that induced neutrophil apoptosis, identifying the presence of markers of apoptosis, and testing treatments that reduce apoptosis. The molecule CD24 displayed reduced expression in cells from sepsis patients compared to those from healthy controls. According to the authors, this finding may explain the reduction in neutrophil apoptosis, as the presence of CD24 results in mitochondrial membrane rupture via induction of reactive oxygen species (ROS)-derived NADPH oxidase (21). The levels of the Fas molecule were higher in sepsis patients, and this finding was associated with reduced apoptosis of circulating neutrophils in sepsis and post-trauma patients (22).

In addition to analyzing the role of PD-L1 in cellular migration, Wang JF et al. (13) also highlighted that sepsis may induce an increase in lymphocytic apoptosis through PD-L1-mediated contact with neutrophils. The same phenomenon of a reduced apoptotic profile was observed in human neutrophils collected from patients with late-stage sepsis, the immunosuppressive stage of this syndrome (17). Finally, in another study that evaluated apoptosis in patients who were treated with vitamin C, caspase-3 levels were lower than those in patients receiving a placebo (23).

#### Phagocytic activity

In this review, phagocytosis was evaluated by only three studies (15%) (11,17,24). The findings were consistent in two studies regarding the reduction of phagocytic capacity in sepsis patients, and the remaining study did not detect phagocytic capacity.

Gurlevik et al. (11) identified G-CSF as a potential inducer of immune function because of its role in stimulating phagocytosis in neutrophils obtained by CLP models injected with G-CSF. According to these authors, the reduction of neutrophil phagocytosis in sepsis patients is associated with a worse prognosis because the reduction was more severe in patients who died (11).

In the analysis of phagocytosis carried out in neutrophils retrieved from sepsis patients, Danikas et al. (24) demonstrated expressive reduction in phagocytosis compared to neutrophils collected from healthy controls. Additionally, patients who survived exhibited upregulated CD64 expression with low phagocytic capacity.

The maintenance of phagocytic ability in patient and healthy control samples was detected in a clinical study published by Demaret et al. (17). In this study, neutrophils were obtained in the immunosuppression stage of sepsis and compared to neutrophils from healthy controls. After analysis of phagocytic activity, no modification of neutrophil activity was observed between sick patients and healthy volunteers.

### Other perspectives of neutrophil activity analysis in sepsis

The remaining six studies included in this review analyzed the presence of molecules potentially associated with neutrophil dysfunction in sepsis, such as pentraxin 3 (PTX3), ROS, and myeloperoxidase (25–28). Part of the studies evaluated the transcription profile of these molecules in blood samples collected from sepsis patients and healthy persons in an attempt to correlate alterations in their levels with clinical prognosis (25–29).

Increased circulating levels of PTX3 were observed in sepsis patients, as were the formation of complexes with neutrophil extracellular traps (NETs) (25). In the latter work, PTX3 was highlighted as a possible diagnostic target on the grounds that this molecule was associated with the detection of high concentrations of extracellular components of neutrophils in sepsis patients.

Two studies evaluated the generation of ROS in samples from patients with sepsis (26,29). One of the studies reported that sepsis patients presented high levels of ROS in peripheral blood samples and that the sustained high levels of these molecules was associated with worse clinical outcomes (26).

The other study evaluated ROS levels by stimulating neutrophils with lipopolysaccharides (LPS), fMLP chemotactic peptide, and *S. aureus* (29). The main finding of this study was that ROS production was reduced in severe sepsis after neutrophil stimulation, with an observed association between the participation of neutrophils and monocytes in oxidative metabolism. Lastly, this same study showed that a SOFA score of 7 was correlated with oxidative metabolism and functioned as a cut-off for patients who progressed to either death or survival (29).

Kothari et al. (27) showed that myeloperoxidase levels were high in samples from patients with sepsis and systemic inflammatory response syndrome (SIRS) and low in patients with septic shock compared to healthy controls.

Lastly, one study addressed the role of the transcriptional response in the plasma of sepsis patients in predicting sepsis severity (28). The authors showed that neutrophils display variable responses in sepsis patients, and several genes were coupled to the immune response in these patients. Additionally, granulocytes showed better sensitivity to immunostimulatory factors identified in the plasma of sepsis patients (28).

## Discussion

Phagocytosis of microbial particles and destruction of infectious agents by enzymes present in intracellular granules are the major functions of neutrophils (4). During inflammation, neutrophils are the first cells to cross the vascular epithelium and reach the site of infection, a process mediated by the interaction between neutrophils and the endothelium, which facilitates rolling, adhesion, and transmigration (4). In this systematic review of 19 studies on neutrophil activity in sepsis, three main mechanisms of neutrophil function were shown to be altered in sepsis patients or animal models of sepsis, namely, migration, phagocytosis, and apoptosis. Furthermore, several circulating molecules that take part in the inflammatory process in sepsis, including PTX3, showed altered levels in blood samples from patients with this syndrome.

In sepsis, neutrophils are not able to reach the infectious agent owing to their reduced migration capacity, a consistent observation across analyzed studies. Further, it is known that microbial control is also altered; however, it remains unclear whether phagocytosis is diminished or preserved.

Based on the studies analyzed, the host defense carried out by neutrophils in sepsis may be stimulated by molecules such as G-CSF or the expression of specific receptors such as CD64. According to Danikas et al. (24), the phagocytic activity in severe sepsis patients is reduced, and the intensity of this dysfunction is proportional to the severity of the infection. However, the correlation between reduced phagocytic activity and the severity of sepsis was not replicated in all studies evaluating this process (11,17).

During sepsis, neutrophils show reduced or impaired migration, with insensitivity to chemotactic stimuli, and correspondingly, the ultimate function of neutrophils in microbial control appears to be less effective. In this context, although migration represents the best-studied neutrophilic function in sepsis, its isolated recovery may not be sufficient to achieve a better prognosis in these patients.

The delay in neutrophilic apoptosis in sepsis was also emphasized. In physiological conditions, these cells show a short life cycle in the bloodstream (i.e., 7 to 10 h). The reduction of the apoptotic process in sepsis patients may denote an adaptation of the body in response to an infectious insult. Nevertheless, there is considerable functional disruption of this cell in severe sepsis. The persistence of these cells for an increased amount of time was not associated with better performance of their functions; on the contrary, this process seems to occur in a dysregulated fashion in sepsis, with cells remaining inert to chemotactic stimuli.

Lastly, we highlight the great number of soluble molecules detected in blood samples from patients or experimental models of sepsis, such as interleukin (IL)1-β, IL-4, IL-6, TNFα, C5a, CXCL8, PTX3, nitric oxide, myeloperoxidase, and Fas. The identification of circulating molecules and the correlation between molecules expressed in the neutrophil cellular membrane and neutrophil activity were better elucidated when CXCR1, PD-L1, caspase 3, CCR2, CXCR2, and CD24 were analyzed, although the corresponding intracellular signaling pathways must be further studied as a unified process in response to hyperinflammation. All molecules were well described and analyzed under a fragmented perspective. The major challenge will be unifying these data and analyzing the processes related to neutrophil activity in a multifactorial framework, which may eventually have a clinical impact.

### Limitations

This review was limited by the exclusion of scientific papers that did not assess the descriptors used in the search and the use of only two databases. Additionally, the stipulation of date limits for surveying the literature and identifying relevant articles was also a limiting factor.

### Conclusion

Migration, phagocytosis, and apoptosis profiles in the neutrophilic response in sepsis are altered and are associated with survival. The current challenge is elucidating the neutrophilic response under a unified clinical framework. Based on this, an alternative therapy for sepsis with a focus on the control or improvement of neutrophil function may be attainable.

## Supplementary Material

Click here to view [pdf].

## References

[1] Levy MM, Fink MP, Marshall JC, Abraham E, Angus D, Cook D (2003). SSCM/ESICM/ACCP/ATS/SIS International Sepsis Definitions Conference. Crit Care Med.

[2] Balk RA (2000). Severe sepsis and septic shock. Definitions, epidemiology, and clinical manifestations. Crit Care Clin.

[3] Faix JD (2013). Biomarkers of sepsis. Crit Rev Clin Lab Sci.

[4] Murphy K (2010). Imunobiologia de Janeway [recurso eletrônico]/ Kenneth Murphy; tradução: 7ed. Dados eletrônicos.

[5] Furze RC, Rankin SM (2008). Neutrophil mobilization and clearance in the bone marrow. Immunology.

[6] Alves-Filho JC, Spiller F, Cunha FQ (2010). Neutrophil paralysis in sepsis. Shock.

[7] Alves-Filho JC, de Freitas A, Spiller F, Souto FO, Cunha FQ (2008). The role of neutrophils in severe sepsis. Shock.

[B08] Alves-Filho JC, Freitas A, Souto FO, Spiller F, Paula-Neto H, Silva JS (2009). Regulation of chemokine receptor by Toll-like receptor 2 is critical to neutrophil migration and resistance to polymicrobial sepsis. Proc Natl Acad Sci USA.

[B09] Rios-Santos F, Alves-Filho JC, Souto FO, Spiller F, Freitas A, Lotufo CM (2007). Down-regulation of CXCR2 on neutrophils in severe sepsis is mediated by inducible nitric oxide synthase-derived nitric oxide. Am J Respir Crit Care Med.

[10] Benjamim CF, Ferreira SH, Cunha FQ (2000). Role of nitric oxide in the failure of neutrophil migration in sepsis. J Infect Dis.

[11] Gurlevik G, Yanikkaya G, Gurleyik E, Ozturk E, Dulundu E, Saglam A (2007). Effects of granulocyte-colony stimulating factor on the polymorphonuclear leukocyte activity and course of sepsis in rats with experimental peritonitis. Surg Today.

[12] Martin EL, Souza DG, Fagundes CT, Amaral FA, Assenzio B (2010). Phosphoinoside-3 kinase γ activity contributes to sepsis and organ damage by altering neutrophil recruitment. Am J Respir Crit Care Med.

[13] Wang JF, Li JB, Zhao YJ, Yi WJ, Bian JJ, Wan XJ (2015). Up-regulation of Programmed cell death 1 ligand 1 on neutrophils may be involved in sepsis-induced immunosuppression. Anesthesiology.

[B14] Schmidt EP, Yang Y, Janssen WJ, Gandjeva A, Perez MJ, Barthel L (2012). The pulmonary endothelial glycocalyx regulates neutrophil adhesion and lung injury during experimental sepsis. Nat Med.

[15] Souto FO, Alves-Filho JC, Turato WM, Auxiliadora-Martins M, Basile-Filho A, Cunha FQ (2010). Essential role of CCR2 in neutrophil tissue infiltration and multiple organ dysfunction in sepsis. Am J Respir Crit Care Med.

[16] Xu R, Lin F, Bao C, Huang H, Ji C, Wang S (2016). Complement 5a receptor-mediated neutrophil dysfunction is associated with a poor outcome in sepsis. Cell Mol Immunol.

[17] Demaret J, Venet F, Friggeri A, Cazalis MA, Plassais J, Jallades L (2015). Marked alterations of neutrophil functions during sepsis-induced immunosuppression. J Leukoc Biol.

[18] Blom C, Deller BL, Fraser DD, Patterson EK, Martin CM, Young B (2015). Human severe sepsis cytokine mixture increases β2-integrin-dependent polymorphonuclear leukocyte adhesion to cerebral microvascular endothelial cells in vitro. Crit Care.

[19] Ubagai T, Nakano R, Kikuchi H, Ono Y (2014). Gene expression analysis of trem1 and grk2 in polymorphonuclear leukocytes as the surrogate biomarkers of acute bacterial infections. Int J Med Sci.

[20] Berger C, Rossaint J, Aken HV, Westphal M, Hahnenkamp K, Zarbock A (2014). Lidocaine reduces neutrophil recruitment by abolishing chemokine-induced arrest and transendothelial migration in septic patients. J Immunol.

[21] Parlato M, Souza-Fonseca-Guimaraes F, Philippart F, Misset B, Captain Study Group, Adib-Conquy M (2014). CD24-triggered caspase-dependent apoptosis via mitochondrial membrane depolarization and reactive oxygen species production of human neutrophils is impaired in sepsis. J Immunol.

[22] Paunel-Görgülü A, Flohé S, Scholz M, Windolf J, Lögters T (2011). Increased serum soluble Fas after major trauma is associated with delayed neutrophil apoptosis and development of sepsis. Crit Care.

[23] Celma IF, Mansilla A, Hassan L, Navarro AG, Comino AM, Bueno P (2009). Effect of vitamin C administration on neutrophil apoptosis in septic patients after abdominal surgery. J Surg Res.

[24] Danikas DD, Karakantza M, Theodorou GL, Sakellaropoulos GC, Gogos CA (2008). Prognostic value of phagocytic activity of neutrophils and monocytes in sepsis. Correlation to CD64 and CD14 antigen expression. Clin Exp Immunol.

[25] Daigo K, Yamaguchi N, Kawamura T, Matsubara K, Jian S, Ohashi R (2012). The proteomic profile of circulating pentraxin 3 (PTX3) comples in sepsis demonstrates the interaction with azurocidin 1 and other components of neutrophil extracellular traps. Mol Cell Proteomics.

[26] Santos SS, Brunialti MKC, Rigato O, Machado FR, Silva E, Salomao R (2012). Geration of nitric oxide and reactive oxygen species by neutrophils and monocutes from septic patients and association with outcomes. Shock.

[27] Kothari N, Keshari RS, Bogra J, Kohli M, Abbas H, Malik A (2011). Increased myeloperoxidase enzyme activity in plasma is an indicator of inflammation and onset of sepsis. J Crit Care.

[28] Khaenam P, Rinchai D, Altman MC, Chiche L, Buddhisa S, Kewcharoenwong C (2014). A transcriptomic reporter assay employing neutrophis to measure immunogenic activity of septic patients' plasma. J Transl Med.

[29] Martins PS, Brunialti MK, Martos LS, Machado FR, Assunção MS, Blecher S (2008). Expression of cell surface receptors and oxidative metabolism modulation in the clinical continuum of sepsis. Crit Care.

